# Development of a plasmid free CRISPR-Cas9 system for the genetic modification of *Mucor circinelloides*

**DOI:** 10.1038/s41598-017-17118-2

**Published:** 2017-12-01

**Authors:** Gábor Nagy, Csilla Szebenyi, Árpád Csernetics, Amanda Grace Vaz, Eszter Judit Tóth, Csaba Vágvölgyi, Tamás Papp

**Affiliations:** 1 MTA-SZTE Fungal Pathogenicity Mechanisms Research Group, Hungarian Academy of Sciences - University of Szeged, Közép fasor 52, H-6726 Szeged, Hungary; 2 Department of Microbiology, Faculty of Science and Informatics, University of Szeged, Közép fasor 52, H-6726 Szeged, Hungary

## Abstract

*Mucor circinelloides* and other members of Mucorales are filamentous fungi, widely used as model organisms in basic and applied studies. Although genetic manipulation methods have been described for some Mucoral fungi, construction of stable integrative transformants by homologous recombination has remained a great challenge in these organisms. In the present study, a plasmid free CRISPR-Cas9 system was firstly developed for the genetic modification of a Mucoral fungus. The described method offers a rapid but robust tool to obtain mitotically stable mutants of *M*. *circinelloides* via targeted integration of the desired DNA. It does not require plasmid construction and its expression in the recipient organism. Instead, it involves the direct introduction of the guide RNA and the Cas9 enzyme and, in case of homology directed repair (HDR), the template DNA into the recipient strain. Efficiency of the method for non-homologous end joining (NHEJ) and HDR was tested by disrupting two different genes, i.e. *carB* encoding phytoene dehydrogenase and *hmgR2* encoding 3-hydroxy-3-methylglutaryl-CoA reductase, of *M*. *circinelloides*. Both NHEJ and HDR resulted in stable gene disruption mutants. While NHEJ caused extensive deletions upstream from the protospacer adjacent motif, HDR assured the integration of the deletion cassette at the targeted site.

## Introduction

Members of the order Mucorales constitute a remarkable group of filamentous fungi. Some of them have biotechnological importance as producers of extracellular hydrolytic enzymes (primarily of lipases and proteases), organic acids (e.g. lactic, fumaric and malic acids), alcohol and carotenoids^1,2^, others are used in starter cultures of oriental food fermentations^3^. Several species, among others *Rhizopus oryzae* and *Lichtheimia corymbifera*, can be agent of opportunistic human infections, known as mucormycoses, which are the second most frequent filamentous fungal deep mycoses after aspergilloses^4,5^. Besides, certain members of the order, such as *Mucor circinelloides*, *Phycomyces blakesleeanus* and *R*. *oryzae*, are widely used model organisms in genetic and molecular biological studies, among others on light sensing^6^, molecular regulation and signal processes^7,8^, sexual reproduction and differentiation^9,10^, morphogenesis^7,11^, carotenogenesis^1,12^ and pathogenicity^13,14^. Furthermore, *M*. *circinelloides* f. *lusitanicus* used in the present study has been involved in several studies addressed to the role of RNA interference in the fungal cell^15^, mechanism and role of morphological dimorphism^7,16^, production of enzymes, carotenoids and other metabolites^1^ and the genetic and molecular background of the pathogenicity of Mucoral fungi^7,17,18^.

Appropriate tools for genetic manipulation including efficient and reliable methods for genetic transformation are basic requirements of cell biological and molecular studies as well as of strain improvement by genetic and metabolic engineering. Although genetic transformation of different Mucoral fungi has been reported several times, targeted gene disruption, deletion and stable integration of the introduced DNA into the host genome have remained a great challenge in this fungal group (for detailed reviews in this topic, see refs^1,19,20^). Plasmid DNAs introduced into Mucoral fungi generally do not integrate into the genome even if they harbor long sequences homologous with the targeted sites and remain autonomously replicating elements frequently causing mitotic instability of the transformants^21^. Moreover, such plasmids often form high molecular weight concatenated structures, which make difficult the interpretation of the results of the transformation experiments^19–21^. The latter phenomenon is explained by the activity of the non-homologous end joining (NHEJ), which is the dominant mechanism of double-strand break repair over homologous recombination in these fungi^21^. Application of linear DNA fragments to force the integration into the genome may lead to ectopic integration and/or unwanted rearranges, formation of concatemers, excision and recircularization^22,23^. One of the reasons of the frequent application of *M*. *circinelloides* in molecular studies is the experience that this organism is more amenable for genetic manipulation than other Mucoral fungi and several transformation methods has been developed for this fungus^24–26^. However, gene disruption or integration by homologous recombination can hardly be carried out in this fungus too and stable integrative transformants has rarely been reported^23^. The difficulties to achieve homologous recombination hampers the construction of mutant libraries, characterization of gene functions, examination of the pathogenicity or performance of recombinant strain improvement studies.

The CRISPR-Cas9 system is a new and versatile genome editing system, which has already been used to target and disrupt genes in some filamentous fungi and yeast, for example in *Aspergillus* strains^27,28^, *Trichoderma reseii* ^29^, *Saccharomyces cerevisiae* ^30–32^, *S*. *boulardii* ^33^, *Candida albicans* ^34^, *C*. *glabrata* ^35^, *Cryptococcus neoformans* ^36^ and *Penicillium chrysogenum* ^37^. Different methods are available for the CRISPR-Cas9 based genome editing in filamentous fungi (for the state of the art achievements in this field, see the review of Shi *et al*.^38^). The *in vivo* expression strategies involve the construction of and transformation with one or two plasmids, which contain the *cas9* gene and the sequences encoding the elements of the guide RNA (gRNA), i.e. the CRISPR-RNA (crRNA) and the trans-activating crRNA (tracrRNA), as well as the protospacer fragment and assure their expression. Although these approaches work well, application of expression vectors for CRISPR-Cas9 genome editing has some drawbacks. Besides that, construction of the corresponding plasmid expressing the *cas9* and the appropriate gRNA can be time consuming and laborious, these systems allow the subsistence and replication of the plasmids after the genome edition event, which can narrow the applicability of the technique and the resulting mutants. For example, such mutants may harbor bacterial antibiotic resistance genes and other bacterial sequences used to select the plasmids during the construction, which is not allowed in many applications. Moreover, long time presence of plasmids and foreign DNA may cause off-target effect as well as unwanted reorganizations and degradations of the transferred DNA and the genome, which have been observed frequently in case of Mucoral fungi^19,20,23^.

In a plasmid free strategy, a ribonucleoprotein (RNP) complex formed from the Cas9 enzyme and the *in vitro* transcribed gRNA is introduced into the cells to edit the targeted gene. Genome editing with RNP formation has been described in different human, animal and plant cell types and it was successfully used to modify the genome of *P*. *chrysogenum* ^37,39–44^. With this method the off-target effect is avoidable but the RNP can be degraded easily^43^.

Therefore, in this study, a CRISPR-Cas9 system without *in vitro* RNP formation and the using of plasmids has been developed and demonstrated as a tool for site-specific mutagenesis of *M*. *circinelloides*. The developed mutation strategy involves the introduction of only the gRNA, i.e. the duplex of the crRNA and the tracrRNA, into the fungal cells to guide the CRISPR-associated Cas9 nuclease and achieves RNA-guided double-strand breaks at the targeted DNA sequences. This plasmid free system has several advantages over the *in vivo* strategy; for example, no additional cloning steps are needed, the off-target effect can be avoided because of the transient exposure of Cas9 in the transformed cells and Cas9 can be active immediately after its uptake by the cells^37^.

To test this approach, two different genes of *M*. *circinelloides*, *carB* and *hmgR2* encoding the enzymes phytoene dehydrogenase and 3-hydroxy-3-methylglutaryl-CoA (HMG-CoA) reductase, respectively, were disrupted and the mutants were characterized. HMG-CoA reductase is a key enzyme of the mevalonic acid pathway of terpene biosynthesis whereas phytoene dehydrogenase participates in the specific β-carotene biosynthesis, which can be considered as a side branch of the general mevalonic acid pathway.

## Results

### Disruption of the *carB* gene in *M*. *circinelloides* by NHEJ

**Figure 1:**
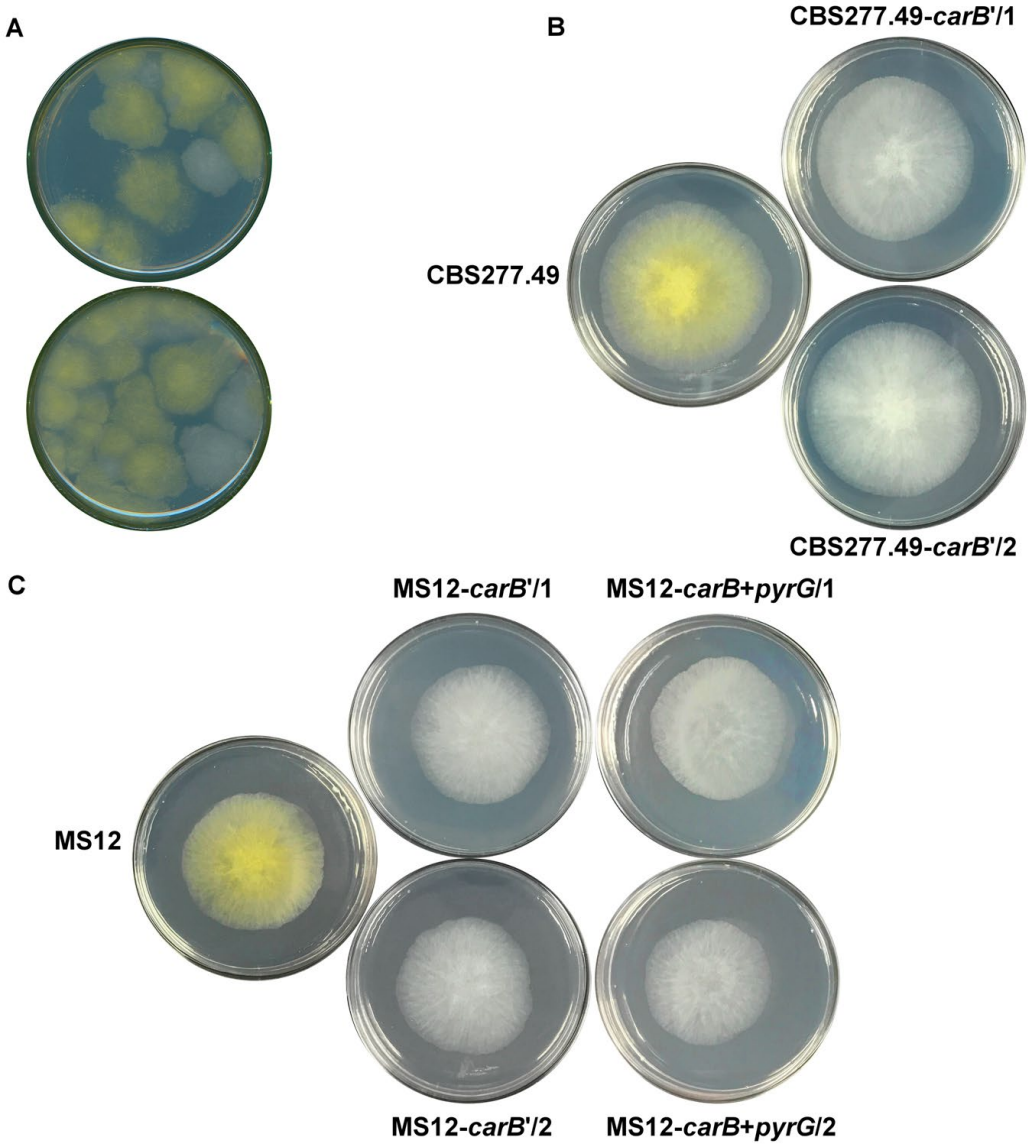
Colonies of *Mucor circinelloides carB* disruption mutants obtained by the CRISPR-Cas9 method. Panel A, selection of the transformed colonies obtained from the wild-type strain, CBS227.49 using NHEJ mutagenesis based on their white colony color. Panel B, colony color of the strain CBS227.49 and its *carB*′ disruption mutants (CBS227.49-*carB*′/1 and CBS227.49-*carB*′/2) created by NHEJ mutagenesis. Panel C, colony color of the strain MS12 and its *carB* disruption mutants created by NHEJ (MS12-*carB*′/1 and MS12-*carB*′/2) and HDR (MS12-*carB* + *pyrG*/1 and MS12-*carB* + *pyrG*/2) approaches.

Protoplasts of the carotenoid producing *M*. *circinelloides* strains MS12 and CBS277.49 were co-transformed with the corresponding gRNA and the Cas9 nuclease. Selection of the transformants was based on the white color of their colonies. The original MS12 and CBS277.49 strains have yellow color due to their β-carotene content. Colonies, in which the targeted *carB* gene was disrupted, had white color since they were unable to synthesize β-carotene (Fig. 1A–C).

To examine the effect of the amount of the gRNA and the Cas9 enzyme on the genome editing efficiency, different concentrations of these components ranging from 50 to 200 µM were added to the protoplasts (Table 1). If only 50 µM gRNA and 50 µM Cas9 were applied, no transformant colonies could be obtained. One hundred micromolar gRNA and Cas9, respectively, proved to be the most efficient to generate white colonies (i.e. to disrupt the *carB* gene) while the further increase of the gRNA and Cas9 amounts did not result in higher number of transformants. Thus, 100 µM of these components were used in all further experiments.

**Table 1:** Characteristics and efficiency of the CRISPR-Cas9 mediated NHEJ and HDR experiments in the tested *M*. *circinelloides* strains.

Host strain	Target	Elements used for transformation^a^	Repair mechanism	Selection method	Transformation frequency (per 10^5^ protoplasts)	Targeting efficiency^b^
*M*. *circinelloides* CBS277.49	*carB*	Cas9 (50 µM), gRNA (50 µM)	—	white phenotype	0	—
*M*. *circinelloides* CBS277.49	*carB*	Cas9 (100 µM), gRNA (100 µM)	NHEJ	white phenotype	2 × 10^4^	100%
*M*. *circinelloides* CBS277.49	*carB*	Cas9 (200 µM), gRNA (200 µM)	NHEJ	white phenotype	1.5 × 10^4^	100%
*M*. *circinelloides* MS12	*carB*	Cas9 (50 µM), gRNA (50 µM)	—	white phenotype	0	—
*M*. *circinelloides* MS12	*carB*	Cas9 (100 µM), gRNA (100 µM)	NHEJ	white phenotype	1.25 × 10^4^	100%
*M*. *circinelloides* MS12	*carB*	Cas9 (200 µM), gRNA (200 µM)	NHEJ	white phenotype	1.2 × 10^4^	100%
*M*. *circinelloides* MS12	*carB*	Cas9 (100 µM), gRNA (100 µM), template DNA (5 µg)	HDR	white phenotype, complementation of uracil auxotrophy	2	100%
*M*. *circinelloides* MS12	*carB*	template DNA (5, 10 or 15 µg)	—	white phenotype, complementation of uracil auxotrophy	0	0
*M*. *circinelloides* MS12	*carB*	Cas9 (100 µM), gRNA (100 µM)	—	white phenotype, complementation of uracil auxotrophy	0	0
*M*. *circinelloides* MS12	*hmgR2*	Cas9 (100 µM), gRNA (100 µM), template DNA (5 µg)	HDR	complementation of uracil auxotrophy	1	100%
*M*. *circinelloides* MS12	*hmgR2*	template DNA (5, 10 or 15 µg)	—	complementation of uracil auxotrophy	0	0
*M*. *circinelloides* MS12	*hmgR2*	Cas9 (100 µM), gRNA (100 µM)	—	complementation of uracil auxotrophy	0	0

^a^Applied concentrations of the elements used are presented in parentheses. ^b^Targeting efficiency means the percentage of the isolated transformants, in which the mutation occurred in the targeted site.

Using 100 µM gRNA and Cas9, transformation (i.e. disruption) frequencies were found to be 1.25 × 10^4^ and 2 × 10^4^ colonies per 10^5^ protoplasts for MS12 and CBS277.49, respectively (Table 1), among which 9 and 13 colonies were isolated for the further analyses.

**Figure 2:**
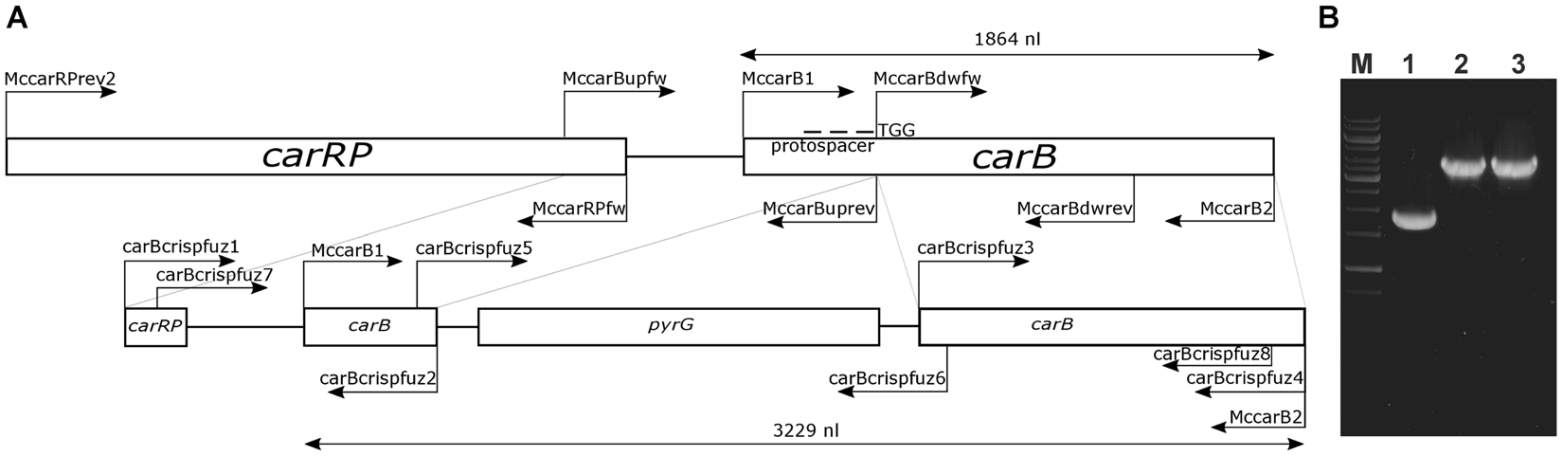
Genome editing strategy designed to disrupt the *carB* gene of *Mucor circinelloides* by the CRISPR-Cas9 method and positions of the primers used in the study (**A**) and PCR analysis of the transformants using the MccarB1 and MccarB2 primers (Table 2) (**B**). TGG indicates the PAM sequence; arrows show the orientations of the primers. Panel B: M, GeneRuler 1 kb DNA ruler (Thermo Scientific); 1, MS12 strain; 2, MS12-*carB* + *pyrG*/1 strain; 3, MS12- *carB* + *pyrG*/2 strain.

To prove the mutation, a region containing the targeted *carB* and the adjacent *carRP* (encoding another carotenogenic enzyme, the phytoene synthase/lycopene cyclase) genes was amplified by PCR from the DNA of the isolated transformants (positions of the primers are presented in Fig. 2A). Sequencing of the PCR products indicated that the mutation occurred at the targeted site in all of the tested transformants. However, extensive deletions (≥2.3 kb) with various lengths upstream from the protospacer, which involved a segment of the *carB* and almost the whole coding sequence of the *carRP* gene, were revealed in each case. Real-time quantitative reverse transcription PCR (qRT-PCR) analysis indicated the lack of *carB* transcripts in the transformants.

To test their mitotic stability, each transformant was passed several times onto selective and non-selective media. All of them proved to be mitotically stable retaining their white phenotype even after 20 cultivation cycles under non-selective growth conditions and the *carB* transcript could also not be detected by qRT-PCR in these colonies.

### Integration of *pyrG* into the *carB* and the *hmgR2* genes by homology directed repair (HDR)

**Figure 3:**
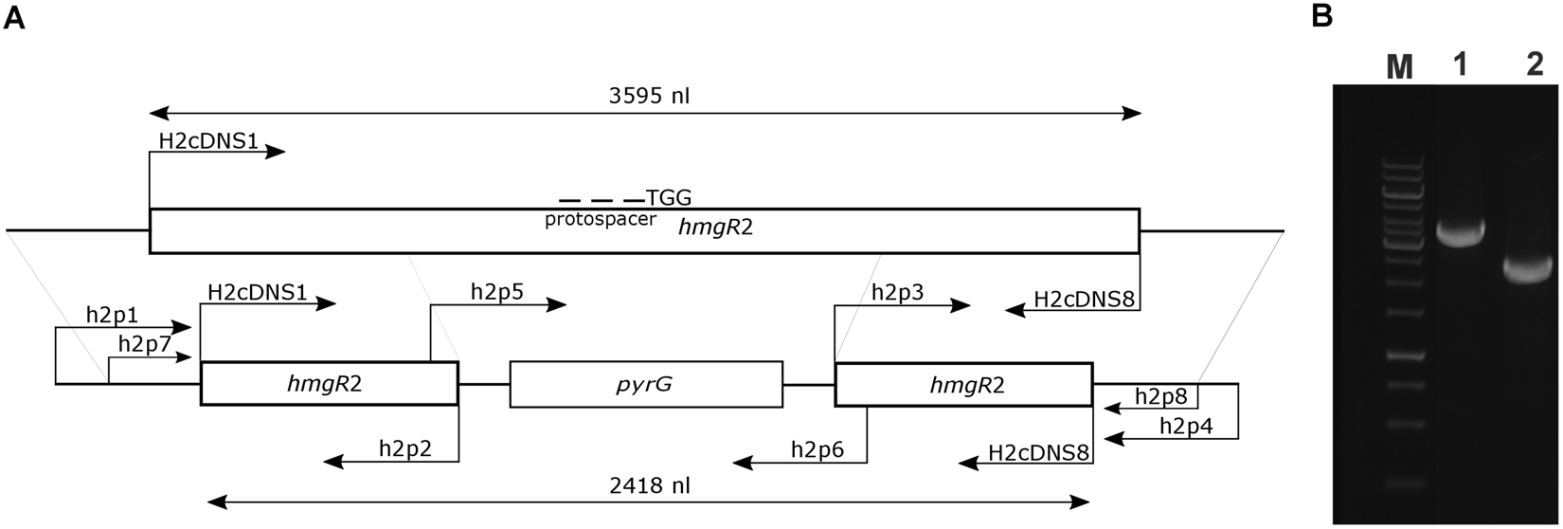
Genome editing strategy designed to disrupt the *hmgR2* gene of *Mucor circinelloides* by the CRISPR-Cas9 method and positions of the primers used in the study (**A**) and PCR analysis of the transformants using the H2cDNS1 and H2cDNS8 primers (Table 2) (**B**). TGG indicates the PAM sequence; arrows show the orientations of the primers. Panel B: M, GeneRuler 1 kb DNA ruler (Thermo Scientific); 1, MS12 strain; 2, MS12-*hmgR2 + pyrG* strain.

Protoplasts of the *M*. *circinelloides* MS12 strain were co-transformed with the corresponding gRNA, the template DNA and the Cas9 nuclease. Template DNA served as the deletion cassette containing the *pyrG* gene as a selection marker and two fragments homologous to the target site to direct the HDR; *pyrG* encodes orotidine-5′-phosphate decarboxylase and complements the uracil auxotrophy of MS12. Figures 2A and 3A shows the genome editing strategy used in these experiments. Although the template DNAs harbored extensive homologous sequences, exclusive application of them (i.e. without the gRNA and Cas9) in different concentrations did not result in any transformants indicating the very low efficiency of homologous recombination in this fungus (Table 1). As the selection based on the complementation of uracil auxotrophy, control experiments using only the gRNA and the Cas9 without the appropriate template DNA also did not result in transformants (Table 1).

When *carB* was targeted, transformation frequency was two colonies per 10^5^ protoplasts (Table 1). Using the MccarB1 and MccarB2 primers (Table 2), a 3.2-kb fragment (Fig. 2B) was amplified by PCR from the DNA of the transformants designated as MS12-*carB* + *pyrG*/1 and MS12-*carB* + *pyrG*/2. In both cases, sequencing of the PCR products confirmed the disruption of the *carB* gene and the integration of the *pyrG* gene into the targeted site. Lack of transcripts was proven by qRT-PCR. Both transformants proved to be mitotically stable as their genotype indicated in Fig. 2 and, accordingly, their white phenotype (Fig. 1C) could be detected even after 20 cultivation cycles performed under non-selective conditions.

**Table 2:** Primers used in the present study.

Primer	Sequence 5′-3′	Amplified DNA
**Phusion PCR for** ***carB*** **disruption**		
carBcrispfuz1	CTGTGCAGGTGCCAGCGCATCACC	*carB* promoter and coding sequence upstream from protospacer
carBcrispfuz2	GGTGGTCTTGAATGCGCTCGTCCAG	
carBcrispfuz3	TGGAGCTGCTGCGATGCGACAAC	*carB* coding sequence downstream from protospacer
carBcrispfuz4	GTCTTGCTCTTCATATGACCAATAG	
carBcrispfuz5	CCGATCTGGACGAGCGCATTCAAGACCACCTGCCTCAGCATTGGTACTTG	*pyrG* with own promoter and terminator sequences
carBcrispfuz6	GTAGTTGTTGTCGCATCGCAGCAGCTCCAGTACACTGGCCATGCTATCG	
carBcrispfuz7	CTTGTGGTAGACAATGTAGTTGTC	phusion pcr product
carBcrispfuz8	CGTCAAAGCTCTCCTGATAAGCCTC	
**Primers for analysis of the** ***carB*** **mutants**		
MccarRPfw	ATGCTGCTCACCTACATGGAAG	*carRP*
MccarRPrev2	TTAAATGGTATTTAGATTTCTCA	
MccarB1	ATGTCCAAGAAACACATTGTCATTA	*carB*
MccarB2	TTAAATGACATTAGAGTTATGAACG	
MccarBupfw	TAGCCAATGACAGCGGTGACGC	upstream region from protospacer
MccarBuprev	GTTGTCGCATCGCAGCAGCTCCA	
MccarBdwfw	TGGAGCTGCTGCGATGCGACAAC	downstream region from protospacer
MccarBdwrev	CGTCAAAGCTCTCCTGATAAGCCTC	
**Phusion PCR for** ***hmgR*** **2 disruption**		
h2p1	ACGGAGCAAGTTTACATTCATACC	*hmgR*2 promoter and coding region upstream from protospacer
h2p2	GATTGGCGGTCTATCATATTTCAG	
h2p3	CTCAACATCTCTTGTACTATGCCC	*hmgR*2 coding sequence downstream from protospacer
h2p4	ATACGTTTGTACCGATGAAGGGA	
h2pyrG5	CTCATTGCTGAAATATGATAGACCGCCAATCGTACAATTCCCTGCCTTCTGGAAG	*pyrG* with own promoter and terminator seqences
h2pyrG6	CGATACAGGGCATAGTACAAGAGATGTTGAGCGCGTGAAAGACCTGCCTTGACTC	
h2p7	TGTGAAATTGGCCTATAATCAGCCT	phusion pcr product
h2p8	GGACAGACATTCCATCGTATACAG	
**Primers for analysis of the** ***hmgR*** **2 mutant**		
h2cdns1	ATGTTGAAAAACGTCAAAAAAGAT	*hmgR*2
h2cdns2	CTATGATTTAATACAACTTCCA	
**Primers used in the qPCR experiment**		
MCactinF	CACTCCTTCACTACCACCGCTGA	117 bp of actin
MCactinR	GAGAGCAGAGGATTGAGCAGCAG	
H2_RT_F	CTCGTATCATCTGTGCCTCTG	107 bp of *hmgR2*
H2_RT_R	AGCAGTGTTACGGTTGTGAG	

In case of the disruption of *hmgR2*, the transformation frequency was only one colony per 10^5^ protoplasts (Table 1). Using H2cDNS1 and H2cDNS2 primers (Table 2), the expected 2.4-kb fragment containing the introduced *pyrG* (Fig. 3B) was amplified by PCR from the transformant designated as MS12-*hmgR2* + *pyrG*. Sequencing also revealed that the CRISPR-Cas9-mediated HDR caused the expected modification (i.e. disruption of the *hmgR2* by the integration of the *pyrG*) in the targeted site. No *hmgR*2 transcripts could be detected by qRT-PCR from this strain and it retained the integrated fragment and proved to be mitotically stable even after 20 cultivation cycles.

### Characterization of the disruption mutants

**Figure 4:**
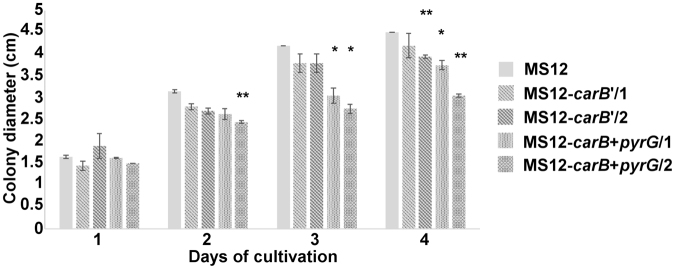
Growth ability of the mutants obtained from the *Mucor circinelloides* MS12 strain at 35 °C. Strains were cultivated on YNB medium for four days under continuous light. Values followed by * and ** significantly differed from the corresponding value of the MS12 strain according to the two-sample t-test; p < 0.05 and p < 0.01, respectively.

Growth ability of the mutants was tested cultivating them on minimal medium (i.e. YNB) at different temperatures for four days in light and measuring the colony diameters daily. At the tested temperatures, significant differences in the growth of the MS12-*hmgR2* + *pyrG* mutant and the original strains were not observed. However, all *carB* disruption mutants showed decreased colony size at 35 °C compared to the MS12 strain (Fig. 4).

All *carB* mutants failed to produce β-carotene and lycopene, while the carotenoid content of the *hmgR2* mutant (457 ± 74 µg/g [dry weight]) did not change significantly compared to that of the original MS12 (476 ± 65 µg/g [dry weight]). In case of MS12-*hmgR*2 + *pyrG*, ergosterol content was also measured. Compared to that of the recipient MS12 strain (4.57 mg/g [dry weight]), it decreased (3.27 mg/g [dry weight]) significantly (p < 0.05) but not completely.

Growth of the different *carB* and *hmgR2* disruption mutants and the MS12 strain under oxidative stress was also examined. Strains, in which *carB* was disrupted, showed no significant changes in their susceptibility to hydrogen peroxide (Supplementary Table S1). At the same time, the strain MS12-*hmgR*2 + *pyrG* displayed reduced growth in the presence of this compound (Supplementary Table S1).

## Discussion

In this study, we transferred the Cas9 enzyme and the gRNA complex into the Mucoral fungus *M*. *circinelloides*, without *in vitro* RNP formation and the using of plasmids to disrupt the *carB* and the *hmgR2* genes. The *carB* and the adjacent *carRP* genes are traditionally recommended for target sites of integration by homologous transformation as their disruption can be easily followed by the white phenotype of the transformant colonies^24,45^. Elements of the corresponding gRNA were simply synthesized by a commercial way and it was introduced into the recipient fungal strain together with the Cas9 enzyme using the polyethylene glycol (PEG)-mediated protoplast transformation technique, which is well established for these fungi^1^. This approach requiring only the *in silico* design of the protospacer sequence and the synthesis of the crRNA and the tracrRNA allows a fast but robust experimental setup and it is well known in genome editing of human and animal cells^46^.

Targeted gene disruption can be achieved by the error-prone NHEJ-mediated mutagenesis generating insertions or deletions (indels) at the site of the strand break caused by the Cas9 enzyme. In this way, *carB* of *M*. *circinelloides* could be successfully disrupted with considerable frequency and the resulting mutants were found to be stable. However, more than 2.3 kb long deletions were detected upstream from the protospacer adjacent motif (PAM) sequence in the resulting mutants. These deletions also affected the adjacent *carRP* gene, which is in the same carotenogenic cluster as *carB* and encodes the phytoene synthase/lycopene cyclase enzyme. Indels induced by NHEJ have most frequently been found to be a few tens of nucleotides long in fungi^27,28^. Thus, the presence of such extensive deletions is unusual and makes difficult the molecular analysis of the NHEJ-generated disruption mutants in *Mucor*. Moreover, it may lead to unwanted consequences, such as disruption of the adjacent gene or genes. Therefore, the application of a template DNA to induce HDR is preferred for the CRISPR-Cas9 mutagenesis of *M*. *circinelloides*.

Double-strand DNA break can increase the frequency of DNA repair by HDR as it was proven in *S*. *cerevisiae* and *C*. *glabrata* ^30,35^. To test and compare the efficiency of this method, we targeted two different genes (i.e. *carB* and *hmgR2*) transferring an appropriate template DNA (functioning as the deletion cassette) together with the gRNA and the Cas9 into the recipient strain. Transformation frequency was relatively low in both cases. However, it should be emphasized that several previous attempts using the classical genetic transformation methods to integrate the same template DNAs into the *Mucor* genome had remained unsuccessful.

HDR resulted in stable transformants, in which gene disruption occurred via the integration of the selection marker at the appropriate sites, indicating the usefulness of this methodology to obtain targeted gene disruption and/or integration in *Mucor*. Stability of the mutants was proven; reorganizations or degradations of the integrated DNA were not found.

Some features of the disrupted mutants were characterized. Apart from the yellow color, morphology of the mutants did not differ from those of the original strains. This lack of morphological consequences of the disruption of carotenoid biosynthesis was also observed in other organisms, such as *Fusarium fujikuroi*, *Neurospora crassa*, *Xanthophyllomyces dendrorhous* or *P*. *blakesleeanus* ^47^. At the same time, mutants showed reduced growth at 35 °C suggesting that carotenoid content has effect on the survival at higher temperatures. Previously, it was also found that carotenoid composition and amount may have a protective effect on *M*. *circinelloides* at higher temperatures^25,48^. In contrast with the fact that β-carotene and carotenoids are effective antioxidants and they are thought to be protective against oxidative stress in fungi^47^, susceptibility of the *carB* mutants to hydrogen peroxide did not change compared to that of the original strains suggesting that carotenoid content has not a significant role in the defense against oxidative stress in *Mucor*, at least against the tested oxidizing agent.

Due to the disruption of *hmgR*2, ergosterol content decreased while the carotenoid content did not change. *M*. *circinelloides* has three *hmgR* genes, i.e. *hmgR1*, *hmgR2* and *hmgR3*, among which *hmgR2* was previously found to be expressed predominantly during the whole life cycle of the fungus^48^. Manipulation of *hmgR* genes has been successfully used to change the carotenoid content in different organisms^49–51^. Previously, overexpression of *hmgR2* and *hmgR3* enhanced the carotenoid production of *M*. *circinelloides* ^48^. The result that disruption of *hmgR2* did not affect the carotenoid content reinforce the overlapping functions of the two genes and the suggestion that *hmgR3* has the major role in the carotenoid biosynthesis.

HMG-CoA reductase activity is thought to affect adaptation to certain environmental challenges, such as osmotic changes, different temperatures or oxidative stress^52–54^, via its role in the biosynthesis of the membrane component, ergosterol and other terpenoids, such as prenyl groups of certain proteins and ubiquinon^55^. Disruption of *hmgR2* gene did not result in reduced or altered the fungal growth at the different temperatures. Previously, transcript abundance of *hmgR2* found to be independent from the different cultivation temperatures^48^. Although ergosterol content decreased after disruption of *hmgR2*, it remained at a considerable level, which also reinforce the overlapping function of the other *hmgR* genes. The strain MS12-*hmgR*2 + *pyrG* displayed reduced growth in the presence of hydrogen peroxide. This feature can be in connection with the decreased ergosterol content. In *S*. *cerevisiae*, low ergosterol level caused sensitivity to paraquat-induced oxidative stress^56,57^. In fact, membrane permeability plays important role in the adaptation to oxidative stress and the altered sterol production may result in higher sensitivity to inducers of oxidative stress^58–60^.

This is the first report of the successful application of the CRISPR-Cas9 system for genetic modification of a Mucoral fungus. Moreover, an approach using directly the guide RNA and the template DNA together with the Cas9 enzyme without the need of plasmid construction was suggested and tested. This technique allows the robust and stable modification (i.e. gene deletion, disruption and/or integration) of *M*. *circinelloides* via the HDR mechanism. Elements of the genome editing system can be easily introduced into the fungus by a simple genetic transformation method, such as the well-developed PEG-mediated transformation. As the described method offers a rapid and reliable genome modification tool to construct mutant strains, it can enhance the various basic and applied studies involving Mucoral strains as model organisms.

## Materials and Methods

### Strains, media and growth conditions

*Mucor circinelloides* f. *lusitanicus* CBS 277.49 (wild type) and MS12 (*leuA* ^−^ and *pyrG* ^−^) strains, were used in this study. The latter strain is auxotrophic for leucine and uracil but wild-type for the carotenoid biosynthesis^48^. For nucleic acid and carotenoid extractions, 10^6^ sporangiospores were plated onto solid minimal medium (YNB; 10 g glucose, 0.5 g yeast nitrogen base without amino acids (Difco), 1.5 g (NH_4_)_2_SO_4_, 1.5 g sodium glutamate and 20 g agar per liter) supplemented with leucine and/or uracil (0.5 mg/ml) if required. Fungal cultures were grown for 4 days under continuous light at 25 °C. To examine the effect of the temperature on the growth of the mutants, fungal strains were cultivated on solid YNB at 20, 25, 30 and 35 °C and the number of the plated spores was 10^4^. To test the mitotic stability of the transformants, malt extract agar (MEA; 10 g glucose, 5 g yeast extract, 10 g malt extract and 20 g agar per liter) was used as a complete, i.e. non-selective, medium.

### Molecular techniques, gRNA and construction of the deletion cassette

Genomic DNA and total RNA were isolated using the ZR Fungal/Bacterial DNA MiniPrep (Zymo Research) and the Direct-zol RNA MiniPrep (Zymo Research) kits, respectively, according to the instructions of the manufacturer. PCR products were isolated and concentrated using Zymoclean Large Fragment DNS Recovery Kit (Zymo Research) and DNA Clean & Concentrator-5 (Zymo Research). To design the oligonucleotide sequences used in the study sequence data available in the *M*. *circinelloides* CBS277.49v2.0 genome database (DoE Joint Genome Institute; http://genome.jgi-psf.org/Mucci2/Mucci2.home.html ^61^) were used. The protospacer sequences designed to target the DNA cleavage in the *carB* and *hmgR*2 gene (CBS277.49v2.0 genome database ID: Mucci1.e_gw1.1.244.1) was the following: 5′-gagcgcattcaagaccacct-3′and 5′-ctctgatatcgtacgcccct-3′ which corresponds to the fragment between the nucleotide positions 220 and 239 downstream from the start codon of *carB* gene and 2117 and 2137 downstream from the start codon of *hmgR*2. Using these sequences, the Alt-R CRISPR crRNA and Alt-R CRISPR-Cas9 tracrRNA molecules were purchased from IDT. To form the crRNA:tracrRNA duplexes (gRNAs), the Nuclease-Free Duplex Buffer (IDT) was used according the instructions of the manufacturer. Deletion cassette also functioning as a template DNA for HDR was constructed by PCR using the Phusion Flash High-Fidelity PCR Master Mix (Thermo Scientific). In case of *carB* gene, at first, two fragments 992 and 986 nucleotides upstream and downstream from the protospacer sequence, respectively, and the *M*. *circinelloides pyrG* gene (CBS277.49v2.0 genome database ID: Mucci1.e_gw1.3.865.1) together with its promoter and terminator sequences were amplified using the primers listed in Table 2. The amplified fragments were fused in a subsequent PCR using the nested primers carBcrispfuz7 and carBcrispfuz8 (Table 2); the ratio of the fragments in the reaction was 1:1:1. The structure of the 3.7-kb fusion product constituting the deletion cassette and location of the primers are presented in Fig. 2A. To disrupt *hmgR*2 two fragment 1817 and 1698 nucleotides upstream and downstream from the protospacer sequence were fused also with *pyrG* in a subsequent PCR using nested primers h2p7 and h2p8 (Table 2).

### qRT-PCR analysis

Transcript level of the *carB* and *hmgR2* genes were analyzed by using the real-time qPCR technique. Reverse transcription reactions were carried out with the Maxima H Minus First Strand cDNA Synthesis Kit (Thermo Scientific) using random hexamer and oligo(dT)18 primers, following the instructions of the manufacturer. The qPCR experiments were performed in a CFX96 real-time PCR detection system (Bio-Rad) using the Maxima SYBR Green qPCR Master Mix (Thermo Scientific) and the primers presented in Table 2. The relative quantification of the copy number and the gene expression was achieved with the 2^−ΔΔCt^ method using^62^ the actin gene (scaffold_07: 2052804-2054242) of *M*. *circinelloides* as a reference. Experiments were performed in biological and technical triplicates.

### Transformation

Deletion cassette, guide RNA and Cas9 enzyme (Alt-R S.p. Cas9 Nuclease, IDT) were introduced together into the *M*. *circinelloides* MS12 strain by PEG-mediated protoplast transformation according to van Heeswijck and Roncero^63^. Protoplasts were prepared as described earlier^64^. In a transformation reaction, 5, 10 or 15 µg template DNA (deletion cassette), 50, 100 or 200 µM gRNA and 50, 100 or 200 µM Cas9 nuclease were added to the protoplasts. If the double-strand break was expected to be repaired by NHEJ instead of HDR, only the gRNA and the Cas9 were added to the protoplasts of the MS12 or the CBS CBS277.49 strain in the abovementioned amounts. Transformants were selected on solid YNB medium by the white color of the transformant colonies due to the disruption of the *carB* gene and/or the complementation of the uracil auxotrophy of the MS12 strain due to the expression of the *pyrG* gene. As a control, the transformation procedure was also carried out on the protoplasts without adding any transforming agents (i.e. the template DNA and the elements of the CRISPR-Cas9 system) in all cases. None of these control experiments resulted in transformant colonies.

### Hydrogen peroxide susceptibility tests

Sensitivity of the fungal strains to menadione and H_2_O_2_ was examined in a 96-well microtiter plate assay. H_2_O_2_ were dissolved in liquid YNB media to prepare stock solutions. Final concentrations of H_2_O_2_ in the wells ranged from 0 to 0.5 mM; chemicals were diluted with liquid YNB medium. Inocula were prepared also in liquid YNB and the final amount of the sporangiospores in the wells was set to 10^4^. Plates were incubated for 48 h at 25 °C and the optical density of the fungal cultures was measured at 620 nm using a Jupiter HD plate reader (ASYS Hitech). The uninoculated medium was used as the background for the calibration and fungal growth in the chemical-free medium was considered as 100%; all experiments were performed in triplicates.

### Analysis of the carotenoid and ergosterol content

Carotenoid extraction was performed as described earlier^65^. For HPLC analysis of the extracts, dried samples were re-dissolved in 100 μL tetrahydrofuran supplemented with butylated hydroxytoluene (100 μg/mL) and 2 μL was subjected onto a Prodigy ODS-3 (4.6 × 150, ODS 3 μm) column (Phenomenex). Separation was carried out using a modular Shimadzu low-pressure gradient HPLC system as described previously^23^. A β-carotene lycopene standards (Sigma) were used for identification and quantification. Analysis and extraction of ergosterol were performed as described earlier^48^.

### Data availability

The authors declare that all data supporting the findings of this study are available within the article and its Supplementary Information file, or are available from the corresponding author upon request.

## Electronic supplementary material

Supplementary Table S1
