# Supplementary material for: Development of a plasmid free CRISPR-Cas9 system for the genetic modification of Mucor circinelloides
Source: Sci Rep. 2017 Dec 1;7:16800. doi: 10.1038/s41598-017-17118-2 (PMC5711797; doi:10.1038/s41598-017-17118-2)
Supplement: Supplementary file 1 — Supplementary Table S1 [file 41598_2017_17118_MOESM1_ESM.pdf]

Development of a plasmid free CRISPR-Cas9 system for the genetic modification of *Mucor circinelloides*

Gábor Nagy[1], Csilla Szebenyi[1,2], Árpád Csernetics[1,2], Amanda Grace Vaz[1,2], Eszter Judit Tóth[1,2], Csaba Vágvölgyi[2] and Tamás Papp[1,2*]

[1] MTA-SZTE Fungal Pathogenicity Mechanisms Research Group, Hungarian Academy of Sciences - University of Szeged, Közép fasor 52., H-6726 Szeged, Hungary

[2] Department of Microbiology, Faculty of Science and Informatics, University of Szeged, Közép fasor 52., H-6726 Szeged, Hungary

Supplementary Table S1. Susceptibility of the *carB'* mutant and the original strains to hydrogen peroxide.

	concentration of hydrogen peroxide (mM)										
Strains	0.5	0.45	0.4	0.35	0.3	0.25	0.2	0.15	0.1	0.05	0
MS12	94%	98%	103%	99%	100%	93%	101%	102%	102%	102%	100%
MS12-*carB*'/1	96%	90%	94%	95%	98%	96%	102%	100%	100%	99%	100%
MS12-*carB*'/2	97%	94%	96%	105%	98%	98%	99%	106%	100%	96%	100%
MS12-*carB+pyrG*/1	98%	94%	103%	99%	104%	99%	99%	103%	103%	98%	100%
MS12-*carB+pyrG*/2	99%	90%	98%	96%	96%	97%	98%	104%	103%	96%	100%
CBS277.49	101%	97%	100%	100%	101%	101%	103%	100%	99%	95%	100%
CBS277.49-*carB*'/1	102%	99%	98%	99%	99%	95%	101%	99%	97%	99%	100%
CBS277.49-*carB*'/2	93%	91%	94%	96%	94%	94%	99%	100%	101%	99%	100%
